# Invasive pneumococcal disease in adults after the introduction of pneumococcal vaccination: a retrospective study in the metropolitan area of Zagreb, Croatia (2010–2022)

**DOI:** 10.3389/fpubh.2024.1480348

**Published:** 2024-12-04

**Authors:** Rok Čivljak, Karla Draženović, Iva Butić, Marina Kljaković Gašpić Batinjan, Eva Huljev, Ninoslava Vicković, Ivan Krešimir Lizatović, Borna Grgić, Ana Budimir, Andrea Janeš, Ana Nikić Hecer, Tajana Filipec Kanižaj, Vanja Tešić, Mirjana Lana Kosanović Ličina, Karolina Dobrović

**Affiliations:** ^1^University Hospital for Infectious Diseases “Dr. Fran Mihaljević”, Zagreb, Croatia; ^2^University of Zagreb School of Medicine, Zagreb, Croatia; ^3^Tajana Janja Lovnički – Kontent, Family Medicine Practice, Zagreb, Croatia; ^4^University of Zagreb School of Dental Medicine, Zagreb, Croatia; ^5^University Hospital Centre Zagreb, Zagreb, Croatia; ^6^University Hospital “Sveti Duh”, Zagreb, Croatia; ^7^University Hospital Center Sestre Milosrdnice, Zagreb, Croatia; ^8^University Hospital Merkur, Zagreb, Croatia; ^9^Andrija Štampar Teaching Institute of Public Health, Zagreb, Croatia; ^10^University of Rijeka School of Medicine, Rijeka, Croatia; ^11^University Hospital Dubrava, Zagreb, Croatia

**Keywords:** invasive pneumococcal disease, pneumonia, meningitis, primary bacteremia, incidence, mortality, adults, vaccination

## Abstract

**Introduction:**

Invasive pneumococcal disease (IPD) is a severe form of illness caused by *Streptococcus pneumoniae* with high morbidity and mortality rate in the general population, particularly in children <5 years of age, adults ≥65 years of age and the immunocompromised. As known, pneumococcal vaccination lowers the risk of IPD so the aim of this study was to investigate whether the introduction of pneumococcal vaccination has influenced the incidence and mortality of IPD in adults in Croatia.

**Materials and methods:**

A retrospective study was conducted among adult patients (aged ≥18 years) hospitalized due to IPD in the metropolitan area of Zagreb from 1^st^ January 2010 to 31^st^ December 2022. Number of vaccine doses distributed were obtained from the healthcare system.

**Results:**

During the study period, 389 patients were hospitalized, of whom 214 (55.5%) were male. The annual incidence of IPD ranged from 0.6 to 4.1/100,000 population. A total of 185 (47.6%) patients were ≥ 65 years of age and 309 (79.4%) were ≥ 50 years of age. In 331 (85.1%) of the patients, at least one risk factor was identified, with age ≥ 65 years being the most common. Bacteremic pneumonia was the most frequent clinical presentation of IPD (66.3%). Indication for vaccination had 249 patients (64%) but only 11 patients (4.4%) were vaccinated. Also, 64 patients (16.5%) died. Serotype was determined in 233 (59.9%) of the isolates, with serotype 3 being the most frequent (49, 21%), followed by serotype 14 (38, 16.3%) and 19A (15, 6.4%). A total of 180 isolates (77.3%) were included in the 13-valent conjugate vaccine, 208 (89.3%) in the 20-valent conjugate vaccine and 212 (91%) in the 23-valent pneumococcal polysaccharide vaccine.

**Discussion:**

The introduction of pneumococcal vaccination has led to a significant decrease in the incidence and mortality of IPD in adults. To further reduce morbidity and mortality from IPD, it is necessary to increase vaccine coverage in adults, particularly in individuals with risk factors. It may be beneficial to lower the recommended vaccination age from ≥65 to ≥50 years as the substantial difference in the incidence rates of IPD between these age groups was noticed.

## Introduction

*Streptococcus pneumoniae* causes two groups of diseases in humans: invasive and noninvasive. While the noninvasive forms of disease (sinusitis, otitis media and non-bacteremic pneumonia) are more common, the invasive forms (bacteremic pneumonia, meningitis and primary bacteremia) are more severe and in such cases, pneumococcus can be identified in primary sterile body fluids, such as blood, cerebrospinal fluid (CSF) or pleural effusion (1). In the general population, IPD contributes significantly to morbidity and mortality, especially in children <5 years of age, adults ≥65 years of age and the immunocompromised, therefore representing a significant public health problem. According to data from the Centers for Disease Control and Prevention in the United States, over 31,000 cases of IPD were reported in 2017, an incidence of 9.4/100,000 population, of whom over 3,500 (11.3%) persons died (2). In 2018, 24,663 confirmed cases of IPD were reported in the EU/EEA, corresponding to a crude notification rate of 6.4 /100,000 population (3). Primary bacteremia (with no recognizable source) is the most common manifestation of IPD, especially in children ≤2 years of age, who account for approximately 40% of IPD cases (2). In the adult population, bacteremia usually accompanies invasive pneumonia or meningitis. According to a retrospective study conducted at the University Hospital for Infectious Diseases “Dr. Fran Mihaljević” in Zagreb, 9.2% of the adult patients hospitalized for IPD in 2010–2013, prior to the introduction of mandatory vaccination of children, had isolated bacteremia, while 58% of the patients had pneumonia (4).

The incidence of IPD in the population depends on many factors: patient age, comorbidities, vaccination status, serotype, season and geographical location. Incidence is higher in the extreme age groups (children <2 years of age and adults ≥65 years of age), as well as persons with comorbidities (5, 6). In the early 2000s, a study was conducted in Croatia to assess the incidence of IPD in children. According to the results of that study, the annual incidence of IPD in children aged <2 is 36.8/100,000, in children from 2 to 5 years of age 16.3/100,000, and in those >5 years of age 2.9/100,000 (7). However, the incidence of laboratory confirmed IPD cases among Croatian adults during a fifteen-year period (2005–2019) was 1.92/100,000, 2.68/100,000, and 4.45/100,000 in 20–49 year olds, 50–64 year olds, and ≥ 65 year olds, respectively (8).

The incidence of IPD depends on the pneumococcal serotype and vaccination status. With the introduction of the 7-valent pneumococcal conjugate vaccine (PCV7) in the year 2002 and the 13-valent PCV13 in 2010 into the mandatory vaccination program for children in Canada, a decreased incidence in the incidence of pneumococcal serotypes contained in the PCV7 and PCV13 vaccines was observed and as well as an increased incidence in the pneumococcal serotypes contained in the 23-valent pneumococcal polysaccharide vaccine (PPSV23) vaccine and non-vaccine pneumococcal serotypes (9). There is a consistent increase in pneumococcal resistance to beta-lactam antibiotics and macrolides worldwide. In Europe, between 8.4 and 20.7% of clinical isolates are resistant to penicillin and between 14.7 and 17.1% to erythromycin (10).

According to data from 2022, penicillin resistance in Croatia was low (5%) but percentage of isolates susceptible, increased exposure to penicillin were high (18%). Resistance to macrolides is continuously very high for more than 20 years, being highest in 2008 (40%) and lowest in 2022 (24%) (11). The future increase in the antibiotic resistance and treatment of IPD caused by resistant pneumococcal strains is an additional argument for the introduction of pneumococcal vaccination in all age groups, including adults. With the use of conjugated and polysaccharide vaccination throughout the world, a decrease in the incidence of IPD, as well as decreased colonization of the nasopharynx, was recorded (12, 13).

The first pneumococcal vaccine was licensed in 1977, which was replaced in 1983 by the PPSV23 currently in use. Since the polysaccharide vaccine is not sufficiently immunogenic in persons <2 years of age, in the early 2000s a conjugate vaccine was introduced worldwide that also stimulates mucosal immunity. Mucosal immunity prevents the colonization of the nasopharynx and thereby reduces the percentage of carriers, having an impact on collective immunity (12, 13). The first conjugate vaccine, PCV7, was licensed in the United States in 2000. In 2010, it was replaced by the PCV13, which is currently in use (2). In 2022, a new 20-valent PCV was registered, which contains 20 polysaccharides of the most common pneumococcal serotypes (serotypes 1, 3, 4, 5, 6A, 6B, 7F, 8, 9 V, 10A, 11A, 12F, 14, 15B, 18C, 19A, 19F, 22F, 23F and 33F) (14). Until 2019, pneumococcal vaccination was recommended and available for some categories, all persons over 2 months of age with functional or anatomical asplenia, immunocompromised persons and immunocompetent persons with cochlear implants or conditions that lead to the CSF leak and infection. Polysaccharide vaccine was recommended for all persons over 2 years of age with functional or anatomical asplenia, immunocompromised persons and immunocompetent persons with chronic heart, lung, kidney or liver disease, diabetes mellitus, alcoholism, cochlear implant and conditions that lead to CSF leak and infection (15).

Since 2019, mandatory pneumococcal vaccination with PCV10 has been implemented in the Croatian National Immunization Program, but only for children <18 years of age (16). According to the Croatian Health Statistics Yearbook for 2023, primary pneumococcal vaccination coverage in children was 92.4% and revaccination coverage was 90.4% (17). In 2021, the Croatian Institute of Public Health revised the recommendations for the pneumococcal vaccination of the adult population. It is recommended for immunocompetent persons to receive only PPSV23, while immunocompromised and asplenic patients should additionally receive PCV13 (18). For now, there is neither data on the overall national vaccination coverage in adults not the impact of the introduction of vaccination against pneumococcal disease on adults in the Republic of Croatia. The aim of this study was to examine whether the introduction of nonmandatory pneumococcal vaccination until 2019 and the introduction of mandatory vaccination for children from 2019 affected the incidence and mortality of IPD in adults.

## Materials and methods

A cohort retrospective study of adult patients hospitalized for IPD in all the hospitals of the metropolitan area of Zagreb from 1^st^ January 2010, to 31^st^ December 2022, was conducted. The analysis included all the adult patients ≥18 years of age whose disease was etiologically confirmed by the presence of *S. pneumoniae* in primary sterile body fluids. Neither persons younger than 18 years of age nor patients with evidence of pneumococci from nonsterile body fluids were included in the study. Demographic data (age, sex), epidemiological data and risk factors for IPD (excessive alcohol consumption, smoking, cardiovascular disease, diabetes, cerebrovascular diseases, respiratory diseases, autoimmune diseases, malignant diseases, immunodeficiency), data on vaccination status, clinical data and pneumococcal serotypes responsible for IPD were collected from hospital medical records.

Serotyping was done using the Quellung method (Statens Serum Institute, Denmark) (19). Antibiotic susceptibility testing was performed following the EUCAST standard. The antimicrobial susceptibility of *S. pneumoniae* strains was tested using the disk diffusion and gradient test methods. In the pneumococcal strains that were positive on the oxacillin screening disc, minimal inhibitory concentrations (MIC) for penicillin and ceftriaxone were determined using gradient test (E-test, Biomerieux, France). *S. pneumoniae* ATCC 49619 was used as a control strain (20).

The data on the number and types of vaccines used were obtained from the Dr. Andrija Štampar Teaching Institute for Public Health in Zagreb. Statistical analysis was performed using Fisher’s exact test to compare in which of the mentioned variables there are significant differences at the bivariate level (two tailed *p*-value <0.001). All the variables that had a significance level of *p* < 0.100 were entered in a binary logistic regression model for vaccination prediction. All the statistical procedures were performed using the IBM SPSS Statistics version 27.0.1.

The study was approved by the ethics committees of all the participating hospitals, as well as the ethics committee of the School of Medicine, University of Zagreb.

## Results

**Figure 1:**
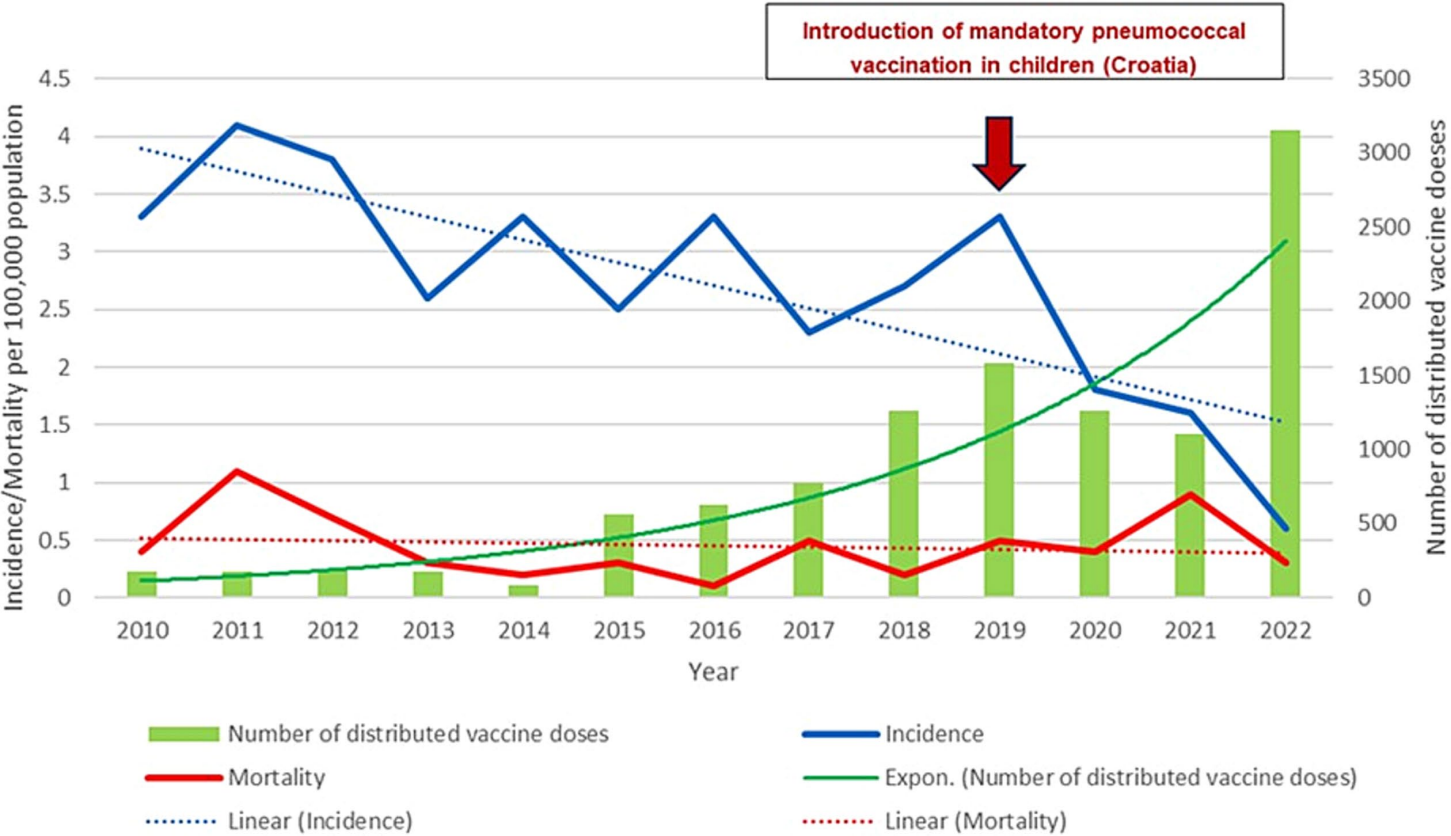
Graphic representation of the annual incidence and mortality of invasive pneumococcal diseases in adult patients in relation to the number of distributed pneumococcal vaccines in the metropolitan area of Zagreb (2010–2022); Linear (Incidence) – Incidence trend; Linear (Mortality) – Mortality trend.

During the study period, 389 patients with confirmed IPD were hospitalized in the metropolitan area of Zagreb. The annual incidences of IPD in adults during the 2010–2022 period are shown in Figure 1. The highest incidence was recorded in 2011 (4.1/100,000 population), and the lowest in 2022 (0.6/100,000 population). The total number of 214 (55.5%) patients were male. The distribution of the subjects according to age group is presented in Table 1. There were 185 (47.6%) persons in the age group of ≥65 years, and in the age group of ≥50 years there were 309 (79.4%). The median age was 63 years (range 25–105 years).

**Table 1:** Basic demographic, epidemiological and clinical data on patients.

Age group (years)	Number of patients *N* (%)	Male *N* (%)	Risk factors			Patients for whom vaccination is indicated*		Case fatality rate *N* (%)
			Without risk factors	One or more risk factors	Two or more risk factors	PCV 13	PPSV23	
18–49	80 (20.6)	53 (66.3)	25 (31.3)	55 (68.8)	26 (32.5)	18 (22.5)	38 (47.5)	9 (11.3)
50–64	124 (31.9)	70 (56.5)	33 (26.6)	91 (73.4)	26 (21.0)	37 (29.8)	77 (62.1)	12 (9.7)
65–74	94 (24.2)	49 (52.1)	0	94 (100)	72 (76.6)	34 (36.2)	66 (70.2)	11 (11.7)
≥ 75	91 (23.4)	42 (46.2)	0	91 (100)	77 (84.6)	25 (27.5)	68 (74.7)	32 (35.2)
Total	389 (100)	214 (55.0)	58 (14.9)	331 (85.1)	229 (58.9)	114 (29.3)	249 (64.0)	64 (16.5)

*They differ according to the type of vaccine and the current recommendations of the Croatian Institute of Public Health.

PCV13–13-valent pneumococcal conjugate vaccine; PPSV – 23-valent pneumococcal polysaccharide vaccine.

Table 2. presents the patient distribution with regard to the presence of individual comorbidities and risk factors. No risk factor was identified in 58 (14.9%) of the patients, while at least one risk factor was identified in 331 patients (85.1%).

**Table 2:** Individual comorbidities and risk factors in adult patients hospitalized for invasive pneumococcal disease in the metropolitan area of Zagreb (2010–2022).

Comorbidities and other risk factors	Subjects *N* (%)
Total number of subjects	389
Without comorbidities/risk factors	58 (14.9)
With ≥1 comorbidity/risk factor^#^	331 (85.1)
With ≥2 comorbidities/risk factors	229 (58.9)
Frequency of individual comorbidities/risk factors	
Age ≥ 65 years	185 (55.9)
Tobacco smoking	96 (29.0)
Chronic cardiovascular disease	77 (23.3)
Diabetes	74 (22.4)
Alcoholism	49 (14.8)
Malignant disease	47 (14.2)
Chronic pulmonary disease	41 (12.4)
Corticosteroid therapy	35 (10.6)
Malignant hematological disease	30 (9.1)
Living in a retirement facility	26 (7.9)
Cerebrovascular disease	26 (7.9)
Chronic liver disease	25 (7.6)
Chronic kidney disease	21 (6.3)
Neurodegenerative disease	18 (5.4)
Trauma of the CNS*	16 (4.8)
Organ transplant	11 (3.3)
Hypogammaglobinemia	7 (2.1)
Congenital or acquired asplenia	5 (1.5)
HIV positive	2 (0.6)

^#Some patients had more than one underlying disease/risk factor.^

*Cerebrospinal fluid leak.

Data on the antibiotic susceptibility testing was available for 326 out of 389 strains (84% of all isolates). Resistance to penicillin was low, <1% (detected in only two of isolates with MIC 3.0 mg/L) while 17% of strains were penicillin susceptible, increased exposure. Susceptibility to ceftriaxone was high, 98% while three isolates were susceptible, increased exposure while three isolates were resistant to ceftriaxone. Resistance to macrolides was high, 24%.

With regard to the clinical presentation of the IPD, 258 (66.3%) had bacteremic pneumonia, 79 (20.3%) had meningitis and 52 (13.4%) had primary bacteremia. The average duration of IPD prior to hospitalization was four days (median 3, range 1–21 days). The patients with isolated bacteremia and those with meningitis were hospitalized on average on the third day of illness, while patients with pneumonia were hospitalized on average on the fourth day of illness. The average duration of hospitalization for patients with IPD was 19 (median 14, range 1–195) days; for patients with primary bacteremia 13.9 days, for patients with bacteremic pneumonia 14.8 days, while for patients with meningitis 33.8 days.

Empiric antimicrobial therapy during hospitalization was initiated with third-generation cephalosporins in 107 (27.5%) of the patients, co-amoxiclav in 48 (12.3%), penicillin in 19 (4.9%) and carbapenems in 11 (2.8%). Combination therapy was started in 204 (52.4%) patients. The most common therapy was combination of third-generation cephalosporin and azithromycin in 87 (22.4%) of the patients, followed by third-generation cephalosporin and vancomycin in 25 (6.4%) and a combination of third-generation cephalosporin and penicillin in 18 (4.6%) The average duration of antimicrobial therapy in patients with IPD was 15 (median 13, range 1–195) days. Due to the severity of IPD, 148 (38%) of the patients were treated in an intensive care unit, of whom 64 (24.8%) had pneumonia, 14 (31.1%) primary bacteremia and 70 (88.6%) had meningitis.

Three hundred twenty-five (83.5%) of the patients with IPD survived, and 64 (16.5%) died. There were 8/79 (10.1%) deaths among the patients with meningitis, 40/258 (15.5%) among the patients with pneumonia, and 16/52 (30.8%) among the patients with primary bacteremia. The largest number of patients hospitalized with IPD (45) was recorded in 2011, while the smallest number (6) was recorded in 2022. The largest number of deaths from IPD (12) was recorded in 2011, while the smallest number (1) was recorded in 2016. There was a decrease in the number of adult patients with IPD according to age, as well as a decrease in the number of deaths during the study period (Figure 1).

**Figure 2:**
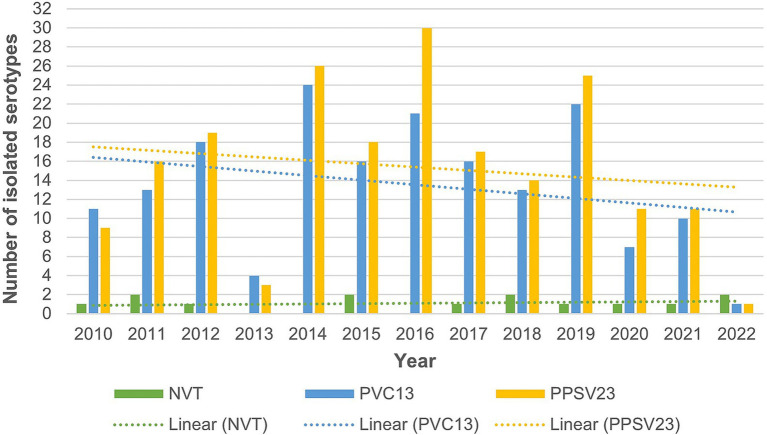
Graphic representation of the distribution of isolated serotypes of pneumococci in adult patients hospitalized due to invasive pneumococcal disease in the metropolitan area of Zagreb according to year (NVT – *non-vaccine serotype*; PVC13–13-valent pneumococcal conjugate vaccines; PPSV23–23-valent pneumococcal polysaccharide vaccine).

The etiological diagnosis of IPD was confirmed by a positive blood culture in 347 (85.1%) of the patients, positive culture from the CSF in 52 (13.4%) patients, positive PCR in the CSF in 38 (9.8%), and a positive culture from pleural effusions in 3 (0.8%) patients. The serotype was determined in 233 (59.9%) of the patients. The frequency of the individual serotypes is presented in Table 3. Of the pneumococcal serotypes confirmed as the causative agent of IPD, 180 (77.3%) were included in the PCV13, 212 (91%) in the PPSV23, and 208 (89.3%) in the 20-valent conjugate pneumococcal (PCV20) (Table 3). The percentages of the individual pneumococcal serotypes were not the same during the period studied. Figure 2 presents the percentages of isolated pneumococcal serotypes according to year. The serotypes are divided into non-vaccine serotypes (NVT), and serotypes contained in the PCV13 and PPSV23. The percentages of the serotypes included in the PCV13 and PPSV23 had a decreasing trend, while the percentages of NVT was unchanged. Table 4. presents the number of patients who died and in whom the pneumococcal serotype had been confirmed. The highest mortality was in patients in whom the serotype 19F (44.4%) and serotype 11A (33.3%) were isolated, followed by patients with the serotype 19A and 11A in whom mortality was 26.7 and 25%, respectively. Figure 1. presents the annual incidence and mortality of IPD in adults hospitalized in the metropolitan area of Zagreb (2010–2022) in relation to the number of people vaccinated with pneumococcal vaccine in said territory during the study period.

**Table 3:** Distribution of individual serotypes of isolated *Streptococcus pneumoniae* in patients with IPD (according to frequency and representation in the PCV13, PPSV23 and PCV20 vaccines).

Sero-type	*N* (%)	Clinical Form of the Invasive Pneumococcal Disease			Serotypes Contained in the Vaccine		
		Sepsis	Meningitis	Pneumonia	PCV13	PCV20	PPSV23
3	49 (21.0)	2	7	40	+	+	+
14	38 (16.3)	2	6	30	+	+	+
19A	15 (6.4)	1	3	11	+	+	+
9 V	13 (5.6)	3	1	9	+	+	+
7F	12 (5.2)	1	1	10	+	+	+
4	12 (5.2)	0	1	11	+	+	+
23F	11 (4.7)	1	0	10	+	+	+
19F	9 (3.9)	3	3	3	+	+	+
1	8 (3.4)	0	0	8	+	+	+
8	7 (3.0)	3	0	4	−	+	+
6B	7 (3.0)	1	1	5	+	+	+
12F	6 (2.6)	2	2	2	−	+	+
11A	6 (2.6)	3	1	2	−	+	+
10A	4 (1.7)	1	2	1	−	+	+
9 N	4 (1.7)	1	0	3	−	−	+
22F	4 (1.7)	0	0	4	−	+	+
18C	4 (1.7)	1	2	1	+	+	+
6C	3 (1.3)	2	1	0	−	−	−
16F	3 (1.3)	1	0	2	−	−	−
15A	2 (0.9)	1	0	1	−	−	−
15C	2 (0.9)	0	1	1	−	−	−
6A	2 (0.9)	0	0	2	+	+	−
23B	2 (0.9)	1	1	0	−	−	−
20	2 (0.9)	1	0	1	−	−	+
17F	1 (0.4)	0	0	1	−	−	+
7A	1 (0.4)	0	0	1	−	−	−
27	1 (0.4)	0	0	1	−	−	−
24F	1 (0.4)	0	0	1	−	−	−
35B	1 (0.4)	0	1	0	−	−	−
33F	1 (0.4)	0	0	1	−	+	−
23A	1 (0.4)	0	1	0	−	−	−
7B	1 (0.4)	0	1	0	−	−	−
Total	233 (100)	31	36	166	180 (77.3)	208 (89.3)	212 (91.0)

*The serotype was identified in 233/389 isolates.

**Table 4:** Distribution of fatal outcomes in patients with invasive pneumococcal disease according to the confirmed serotypes of the *Streptococcus pneumoniae* isolates.

Serotype	Number of isolates	Number of deaths	Case fatality rate (%)
3	49	6	12.2
14	38	2	5.3
19A	15	4	26.7
7F	12	2	16.7
4	12	1	8.3
23F	11	1	9.1
19F	9	4	44.4
1	8	1	12.5
8	7	1	14.3
6B	7	1	14.3
11A	6	2	33.3
12F	6	1	16.7
10A	4	1	25.0
16F	3	2	66.7
6A	2	1	50.0
15C	2	1	50.0
23B	2	1	50.0
15A	2	2	100
17F	1	1	100

Of the 389 patients hospitalized for IPD, 249 (64%) had an indication for pneumococcal vaccination, although only 11 (4.4%) were vaccinated (Table 1).

The polysaccharide vaccine was predominant and the largest number of persons vaccinated, 3,151 (2,910 with PPSV23 vaccine and 241 with PCV13 vaccine) was recorded in the year 2022. Table 5 shows differences in the comorbidities and risk factors among patients hospitalized for invasive pneumococcal disease in the metropolitan area of Zagreb (2010–2022) regarding their pneumococcal vaccination status. Using Fisher’s exact test to detect any significant differences among the variables at the bivariate level, variables whose *p* value was less than 0.100 were used in a binary logistic model to predict of belonging to the group of patients who were vaccinated against pneumococci (Table 6). This regression model is statistically significant (*p* < 0.001) and explains 26.6% of the variance of the dependent variable (vaccination) and correctly classifies 97.4% of the respondents. Statistically significant higher vaccination coverage was shown in a group of patients with hypogammaglobulinemia (OR 15.42; 95% CI 1.70–139.73), congenital or acquired asplenia (OR 76.32; 95% CI 9.45–616.37) and CNS trauma (OR 10.29; 95% CI 1.83–57.76).

**Table 5:** Differences in comorbidities and risk factors among hospitalized patients for invasive pneumococcal disease in the metropolitan area of Zagreb (2010–2022) regarding their pneumoccocal vaccination status.

Comorbidities and risk factors		Pneumococcal vaccination				*P* value
		No		Yes		
		*N*	%	*N*	%	
Gender	Male	207	54.8%	7	63.6%	0.761
	Female	171	45.2%	4	36.4%	
Alcoholism	No	269	71.2%	7	63.6%	0.737
	Yes	109	28.8%	4	36.4%	
Tobacco smoking	No	249	66.0%	7	63.6%	1.000
	Yes	128	34.0%	4	36.4%	
Hypogammaglobulinemia	No	373	98.7%	9	81.8%	**0.014**
	Yes	5	1.3%	2	18.2%	
Congenital or acquired asplenia	No	375	99.5%	8	72.7%	**<0.001**
	Yes	2	0.5%	3	27.3%	
HIV infection	No	376	99.5%	11	100.0%	1.000
	Yes	2	0.5%	0	0.0%	
Malignant hematology diseases	No	350	92.6%	9	81.8%	0.205
	Yes	28	7.4%	2	18.2%	
Organ transplant	No	368	97.4%	10	90.9%	0.274
	Yes	10	2.6%	1	9.1%	
Corticosteroid therapy	No	329	87.0%	9	81.8%	0.643
	Yes	49	13.0%	2	18.2%	
Chronic pulmonary disease	No	337	89.2%	11	100.0%	0.615
	Yes	41	10.8%	0	0.0%	
Heart disease	No	302	79.9%	10	90.9%	0.700
	Yes	76	20.1%	1	9.1%	
Cardiovascular disease	No	202	53.4%	8	72.7%	0.236
	Yes	176	46.6%	3	27.3%	
Diabetes mellitus	No	304	80.4%	11	100.0%	0.134
	Yes	74	19.6%	0	0.0%	
Chronic kidney disease	No	357	94.4%	11	100.0%	1.000
	Yes	21	5.6%	0	0.0%	
Neurodegenerative disease	No	360	95.2%	11	100.0%	1.000
	Yes	18	4.8%	0	0.0%	
Cerebrovascular diseases	No	352	93.1%	11	100.0%	1.000
	Yes	26	6.9%	0	0.0%	
CNS trauma	No	364	96.3%	9	81.8%	**0.070**
	Yes	14	3.7%	2	18.2%	
Chronic liver disease	No	330	87.3%	11	100.0%	0.373
	Yes	48	12.7%	0	0.0%	
Malignant disease	No	320	84.7%	9	81.8%	0.681
	Yes	58	15.3%	2	18.2%	

Bold values are those that are statistically significant.

**Table 6:** Binary logistic regression model of pneumococcal vaccination prediction.

	OR	95% CI		*P*
		Lower	Upper	
Hypogammaglobulinemia	15.42	1.70	139.73	0.015
Congenital or acquired asplenia	76.32	9.45	616.37	<0.001
CNS trauma	10.29	1.83	57.76	0.008

## Discussion

The results of the study demonstrate that the incidence of IPD in the metropolitan area of Zagreb during the study period decreased from 4.1/100,000 population in 2011 to 0.6/100,000 population in 2022. Since 2019 more significant decline in number of IPD was observed what can be explained with introduction of mandatory pneumococcal vaccination into the childhood immunization program (16). Also, during COVID-19 pandemic decrease of IPD was observed in Croatia as reported in other European countries (11). The introduction of the mandatory pneumococcal vaccination of children led to a decrease in the incidence of IPD in other European countries and elsewhere (12, 21). Moreover, the greater vaccination of children resulted in a decrease in the incidence of IPD in the general population (22, 23).

In addition, there was greater vaccination coverage among adults in the period from 2010 to 2022. One of the reasons for the increase in pneumococcal vaccination rates may be the campaign React and Prevent, which was launched in Croatia, with the aim of educating the public about pneumococcal vaccination (24). Generally, developed nations, including the UK, Canada, France, and Germany, have started recommending PCV20 (25–28) However, during the study period PCV20 was not available in Croatia.

Several interventions were associated with increased pneumococcal vaccination coverage in adults, with prioritization of vaccination schemes, primary care interventions and awareness campaigns being the most effective (29). The results of several studies concluded that combining interventions was found to be more successful, but barriers and interventions could vary for certain population subgroups which should be take into account while tailoring programs (30).

Our results demonstrate that during the period studied, the number of deaths due to IPD decreased (from 12 in 2011 to 3 in 2022) as well as mortality (from 0.4/100,000 population in 2010 to 0.3/100,000 population in 2022). A study conducted in Denmark also showed a reduction in mortality (from 3.4/100,000 population prior to the introduction of PCV to 2.4/100,000 after the introduction of PCV13), especially among the unvaccinated population (22). Age is one of the known risk factors for IPD. This study only included adult patients (≥ 18 years of age) but not children, in whose age group the incidence of IPD is also high, especially those under 5 years of age (7). Among our patients, 47.6% were ≥ 65 years of age, but as many as 79.4% were ≥ 50 years of age. This demonstrates that persons younger than 65 years of age also belong to the risk population, due to which perhaps the recommended age for the vaccination of adults should be lowered from 65 to 50 years of age (31–34).

The majority of the subjects (85.1%) had at least one risk factor for developing IPD, while as many as 58.9% had two or more. The highest number of patients with confirmed risk factors were ≥ 65 years of age. A similar finding was recorded in a study conducted in Columbia, where 80.5% of the patients had at least one risk factor, while 37.3% had two or more. Furthermore, the largest number of patients with confirmed risk factors were ≥ 65 years of age (31). In a study conducted in Croatia in 2013, 68.9% of the patients had at least one risk factor (4). Among our study subjects, 64% had indications for pneumococcal vaccination, although only 4.4% were vaccinated. According to the current recommendations of the Croatian Institute of Public Health, among our subjects 64% of the patients had indications for the PPSV23 vaccine and 29.3% for the PCV13. The vaccination of subjects with indications for vaccination against pneumococcal disease varies among countries. According to the results of a study involving nine European countries, including Croatia, the highest pneumococcal vaccination rate (30.5%) was in Spain and the lowest (0.7%) was in Croatia (6). Subjects ≥4 years of age were included in the study. The vaccination rate in the United States is 23.3% among those 19–64 years of age with increased risk for IPD, while there is a somewhat higher (69%) pneumococcal vaccination rate in those ≥65 years of age (35).

Death occurred in 16.5% of the patients: 10.1% among patients with meningitis, 15.5% among patients with bacteremic pneumonia and 30.8% in patients with primary bacteremia. By comparison, in a study conducted in Sweden, death occurred in 9.9% of the participants: 13% among patients with meningitis, 8.3% among patients with pneumonia and 21% among patients with primary bacteremia (36). The most common serotype was serotype 3 detected in 21% of the patients followed by serotype 14 (16.3%) and serotype 19A (6.4%). Among the serotypes detected, 77.3% belonged to the serotypes covered by the PCV13 vaccine, 89.3% were covered by the PCV20 vaccine and 91% by the PPSV23 vaccine. These results demonstrate that the pneumococcal serotypes that caused disease in our patients are well covered by the available vaccines.

In numerous studies, it was stated that the introduction of the PCV7, and later also PCV13, led to a decrease in the number of serotypes covered by the vaccines used but also to an increase in IPD due to NVT. Therefore, there is a need for the constant development of new vaccines that will cover as many pneumococcal serotypes as possible (6, 12, 37). Since 2022 new PCV20 vaccine was available in Croatia. This vaccine contains the pneumococcal serotypes in the PCV13 vaccine plus an additional seven serotypes. Of the 233 pneumococcal isolates serotyped in our study, as many as 89.3% belong to the serotypes contained in the PCV20 vaccine, which would mean that the use of this vaccine in the Republic of Croatia could prevent a large percentage of IPD in adult patients. According to the Centers for Disease Control and Prevention, the PCV20 vaccine is currently recommended for children <5 years of age, adults ≥65 years of age as well as adults 19–64 years of age who have a risk factor (38). If the PCV20 vaccine is administered, neither prior administration of the PPSV23 vaccine nor a booster is needed. As previously mentioned, according to the latest the Centers for Disease Control and Prevention guidelines, vaccination with only the PCV20 vaccine is recommended (38). In Denmark, a study was conducted to compare the benefits of the PCV20 vaccine with those of the PPSV23 vaccine. According to the study results, approximately 75% of the strains are covered by the PPSV23 vaccine, while 65% are covered by the PCV20 vaccine. Through further analysis, they concluded that the administration of the PCV20 vaccine, together with the PPSV23 vaccine or alone, leads to a decreased incidence of IPD and reduced mortality. Moreover, the inclusion of PCV20 vaccine instead of PPSV23 in the National Vaccination Program will also lower the costs (39). Nowadays, more than 100 pneumococcal serotypes have been described, of which 8–10 serotypes are the most common causes of pneumococcal diseases in humans (2). In our study, it was possible to determine the serotype in 59.9% of the isolates with serotypes 3, 14, 19A and 9 V being the most common. In a study conducted during 2010–2013, the serotypes most represented were 3, 1, 19A and 6B (4). In a study conducted among children in Croatia, the most frequently represented serotypes were 14, 6B, 18C and 23F (7). According to other studies, these serotypes are given as the most common in adults and children. All these serotypes are included in all three pneumococcal vaccines for adults (PCV13, PCV20 and PPSV23) currently available in Croatia. The pneumococcal serotype can also affect mortality. In a study conducted in Switzerland, it was demonstrated that mortality from IPD is greater if the infection is caused by the serotypes 3, 19A or 19F (5). In our study, mortality among the most common serotypes 3, 14 and 19A was 12.2, 5.3 and 26.7%, respectively, while serotype 19F had high mortality of 44.4% (4 out 9 patients). In some countries, there has been an increase in the incidence of IPD caused by NVT following the introduction of PCV vaccine in the mandatory children’s vaccination programs (9, 37). According to our research, during the study period there was no increase in the NVT among the causes of IPD in Croatia.

Antibiotic resistance in *Streptococcus pneumoniae,* mainly to penicillin, macrolides and recently flouroquinolones, is recognized as a global health problem. The Croatian network of microbiological laboratories has a tradition of over 25 years in the antibiotic resistance surveillance of the most important human pathogens including *Streptococcus pneumoniae.* Based on our national data, resistance to macrolides and penicillin non-wild type has decreasing trends since 2019 (11). Our study confirms that the main problem regarding resistance in Croatia is high resistance to macrolides and the high percentage of penicillin non-wild type isolates but resistance to penicillin is continuously very low. Therefore, macrolides are not suitable for the treatment of pneumococcal infections in Croatia while amoxicillin is identified as the best choice, which is in line with the European and Croatian guidelines (40, 41).

This study has several limitations. It includes only participants from the metropolitan area of Zagreb, although it covers almost one fourth of the total population of Croatia. Only adult subjects are included in the study, although in order to speak more clearly about the effects of the introduction of mandatory pneumococcal vaccination it would be necessary to include subjects <18 years of age, for whom mandatory pneumococcal vaccination has already been introduced. Due to the facts that pneumococcal conjugate vaccine was introduced in to the Croatian national childhood immunization program in 2019 and our study period included the COVID-19 pandemic period when the incidence of IPD was low, our study did not show a post-vaccinal increase of IPD caused by NVT due to the short follow up period. Therefore, longer monitoring of IPD and serotypes is need for to drawing more thorough conclusions.

In conclusion, the introduction of pneumococcal vaccination led to a decrease in the incidence and mortality from IPD in adults. In order to achieve a further decrease in morbidity and mortality from IPD, it is necessary to increase vaccination coverage among adults, especially in persons with risk factors and also lower the recommended age for vaccination from the existing 65 to 50 years of age. Therefore, prioritization of vaccination schemes, primary care interventions, awareness campaigns and combinations with other preventive interventions are needed to achieve this goal. It is necessary to continue this study and in the coming period analyze the effects of the mandatory vaccination of children on the adult population and the effects of the recommendations for the pneumococcal vaccination of adults.

## Data Availability

The original contributions presented in the study are included in the article/supplementary material, further inquiries can be directed to the corresponding author.
